# Propionic acid induces alterations in mitochondrial morphology and dynamics in SH-SY5Y cells

**DOI:** 10.1038/s41598-023-40130-8

**Published:** 2023-08-15

**Authors:** Erin Buchanan, Caitlyn Mahony, Sophia Bam, Mohamed Jaffer, Sarah Macleod, Asandile Mangali, Mignon van der Watt, Sholto de Wet, Rensu Theart, Caron Jacobs, Ben Loos, Colleen O’Ryan

**Affiliations:** 1 Department of Molecular and Cell Biology, University of Cape Town, Cape Town, 7700 South Africa; 2 Electron Microscope Unit, University of Cape Town, Cape Town, 7700 South Africa; 3 Department of Physiological Sciences, Stellenbosch University, Matieland, Stellenbosch, 7602 South Africa; 4 Department of Electrical and Electronic Engineering, Stellenbosch University, Matieland, Stellenbosch, 7602 South Africa; 5 Department of Pathology, Wellcome Centre for Infectious Diseases Research in Africa and IDM Microscopy Platform, Institute of Infectious Disease and Molecular Medicine, University of Cape Town, Cape Town, 7700 South Africa; 6 Neuroscience Institute, University of Cape Town, Cape Town, 7700 South Africa

**Keywords:** Cellular imaging, Gene expression, Cellular neuroscience

## Abstract

Propionic acid (PPA) is used to study the role of mitochondrial dysfunction in neurodevelopmental conditions like autism spectrum disorders. PPA is known to disrupt mitochondrial biogenesis, metabolism, and turnover. However, the effect of PPA on mitochondrial dynamics, fission, and fusion remains challenging to study due to the complex temporal nature of these mechanisms. Here, we use complementary quantitative visualization techniques to examine how PPA influences mitochondrial ultrastructure, morphology, and dynamics in neuronal-like SH-SY5Y cells. PPA (5 mM) induced a significant decrease in mitochondrial area (*p* < 0.01), Feret's diameter and perimeter (*p* < 0.05), and in area^2^ (*p* < 0.01). Mitochondrial event localiser analysis demonstrated a significant increase in fission and fusion events (*p* < 0.05) that preserved mitochondrial network integrity under stress. Moreover, mRNA expression of *cMYC* (*p* < 0.0001), *NRF1* (*p* < 0.01), *TFAM* (*p* < 0.05), *STOML2* (*p* < 0.0001), and *OPA1* (*p* < 0.01) was significantly decreased. This illustrates a remodeling of mitochondrial morphology, biogenesis, and dynamics to preserve function under stress. Our data provide new insights into the influence of PPA on mitochondrial dynamics and highlight the utility of visualization techniques to study the complex regulatory mechanisms involved in the mitochondrial stress response.

## Introduction

Mitochondria are integral players in diverse cellular functions that extend beyond their canonical roles in energy production and biosynthesis. Mitochondrial metabolism is a key regulator of calcium signaling, metabolic and redox homeostasis, inflammatory signaling, epigenetic modifications, cellular proliferation, differentiation, and programmed cell death^1^. In particular, mitochondrial metabolism is essential for the development, survival, and function of neurons, as well as being widely implicated in diverse manifestations of neuropathology^2–4^.

Over the past decade, metabolic state has become well-established as a central regulator of neurogenesis, differentiation, maturation, and plasticity^5,6^. More recently, mitochondrial morphology and dynamics have emerged as particularly important components of mitostasis, which refers to the dynamic processes that maintain the intracellular pool of healthy mitochondria. Mitochondrial dynamics are modulated by complex, interdependent pathways, ranging from mitochondrial biogenesis and bioenergetics to mitochondrial fission, fusion, transport, and clearance^7,8^. A disruption to any of these integrated mechanisms impairs the maintenance of a healthy mitochondrial network and has profound functional implications for neurodevelopment^9,10^. In fact, dysregulation of mitochondrial dynamics is observed across numerous psychiatric, neurodegenerative, and neurodevelopmental disorders including autism spectrum disorders (ASD)^11,12^.

ASD is a heterogeneous neurodevelopmental disorder with a complex genetic and epigenetic architecture. The heritability of ASD is undisputed, but the underlying molecular etiology remains poorly characterized. Cumulative data from preclinical models, clinical studies, and multi-omic molecular datasets provide increasing evidence for mitochondrial dysfunction in ASD^13,14^. Previously, we conducted a whole-genome DNA methylation screen in an ASD cohort and identified differentially methylated genes that converged on mitochondrial metabolic pathways^15^. Subsequently, we reported differential methylation of central regulators of mitochondrial biogenesis and dynamics, which was associated with elevated mitochondrial DNA copy number and an altered urinary metabolic profile in ASD^16^. Our data contributed to the growing body of evidence that mitochondrial dynamics and homeostasis plays a central role in ASD pathophysiology. Thus, improving the mechanistic understanding of the relationship between mitochondrial dynamics, morphology, and function is a key target for ongoing research into neurological conditions characterized by secondary mitochondrial dysfunction.

Molecular methods are commonly used to investigate the role of specific genes in response to mitochondrial stress. However, this approach can be limited by the multifaceted and temporal nature of the mechanisms that govern mitostasis. Furthermore, the differential expression of mitochondrial genes is an indirect proxy to functional change, especially since only a limited number of genes are generally analyzed. Thus, more direct methods to explore mitochondrial function and bioenergetics have been proposed^17^. Mitochondrial morphology is closely coupled with mitochondrial dynamics; the shape, connectivity, and structure of mitochondria are essential for energy generation as well as mitochondrial- and cellular-survival^5,18^. Moreover, disparate components of mitostasis converge on alterations to mitochondrial morphology, which could function as a useful endpoint of mitochondrial dysfunction to inform subsequent mechanistic studies.

The morphology of mitochondria can be directly visualized by transmission electron microscopy (TEM), which allows for the detailed examination of cellular ultrastructure. TEM yields direct visualization of mitochondrial morphology, shape, and cristae structure at the resolution of single mitochondria as opposed to relying only on gene transcription, protein expression, or parameters of mitochondrial function across cell populations^17,19,20^. Moreover, TEM facilitates the study of interactions between mitochondria and other organelles like the endoplasmic reticulum and autophagosomes, which play a critical role in mitochondrial function and homeostasis^21,22^. Consequently, this makes TEM a good starting point for investigating mitochondrial dysfunction before focusing on specific pathways or genes. As mitochondrial function is increasingly implicated in neuropathology, there is a clear need to be able to directly, and quantitatively, investigate mitochondrial morphology and dynamics in in vitro neuronal models.

In this paper we explore mitochondrial dynamics in a neuronal model for mitochondrial dysfunction in ASD. We previously reported the differential methylation of the Propionyl-CoA carboxylase Beta (PCCB) in ASD^15^, a subunit of the mitochondrial enzyme, Propionyl-CoA carboxylase, PCC. A dysregulation of PCC is known to induce a toxic accumulation of propionyl derivatives, including propionic acid (PPA)^23–25^. PPA has been shown to impair neuronal metabolism and alter behavior in vivo and is an established animal model to study neurodevelopmental mechanisms involved in ASD^26–28^. Moreover, PPA has been reported to disrupt mitochondrial membrane potential, biogenesis, and respiration in vitro and is widely used to model mitochondrial dysfunction in neurons^29,30^*.* However, the effect of PPA-induced mitochondrial dysfunction on mitochondrial morphology and dynamics remains inadequately studied.

This study uses complementary visualization techniques to quantitatively evaluate the effect of PPA on mitochondrial morphology, dynamics, and function in SH-SY5Y cells. First, we established a TEM method to visualize changes to mitochondrial morphology and ultrastructure^17,31,32^. We also used mitochondrial event localiser (MEL) analysis to quantify changes in the balance between fission and fusion events, mitochondrial count, and volume under PPA stress, given the dynamic nature of mitochondria ^33^. Finally, we explore whether mitochondrial morphology and dynamics are associated with alterations in the expression of genes involved in biogenesis, fission, and fusion. Together, our data illustrate the challenges of elucidating the complexity of the regulatory mechanisms involved in mitochondrial dynamics. We emphasize the utility of TEM to explore mitochondrial morphology as a measurable, convergent endpoint of mitostasis in SH-SY5Y cells. Moreover, we highlight how TEM data is most informative in combination with visualization techniques that also capture the dynamic events that respond to metabolic stress. Further characterizing the molecular regulatory mechanisms that maintain mitostasis in neuronal cells has the potential to yield important insights into the mitochondrial component of neurodevelopmental and neurodegenerative disorders.

## Results

### Propionic acid reshapes mitochondrial morphology

**Figure 1:**
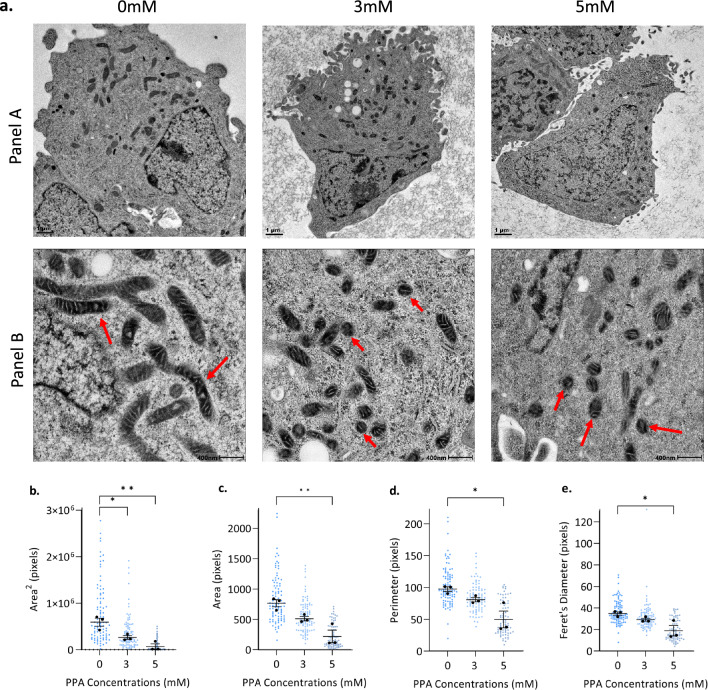
Propionic acid (PPA) reshapes mitochondrial morphology. (**a**) Representative transmission electron microscopy (TEM) images show a decrease in mitochondrial size with mitochondria becoming smaller and rounder with increasing PPA treatment; 0 mM (untreated), 3 mM and 5 mM respectively. Red arrows indicate mitochondria. (**b–e**) SH-SY5Y cells treated with PPA for 24 h underwent preparation for TEM and results were analyzed using Fiji/ImageJ. Four of the eight parameters showed significant differences between control (untreated, 0 mM PPA) and treated (3 mM and 5 mM PPA) cells. (**b**) Area^2^, (**c**) Area, (**d**) Perimeter, (**e**) Feret’s diameter. Significant differences were identified using one-way ANOVA (control compared to treatments) and Dunnett’s test for multiple comparisons (*p* < 0.05). Data points represent mitochondrial averages per individual cell and error bars represent mean ± SEM. The data shown represents n = 3 with a minimum of 24 cells per replicate; a total of 266 images were analyzed; * indicates *p* < 0.05, ** indicates *p* < 0.01.

To induce mitochondrial stress, SH-SY5Y cells were treated with PPA, administered using 3 mM and 5 mM of sodium propionate (NaP). Samples underwent cryogenic sample preparation using high-pressure freezing and freeze-substitution prior to TEM (Fig. 1a). We developed an automated mitochondrial image analysis pipeline to measure eight morphological parameters across mitochondrial populations in three biological repeats. We found that four of these parameters were significantly altered by PPA treatment: area^2^, area, perimeter, and Feret’s diameter (Fig. 1b–e). The area^2^ was significantly decreased at both 3 mM and 5 mM PPA treatment (*p* = 0.0183 and *p* = 0.002, respectively) (Fig. 1b), whilst the area (*p* = 0.003), perimeter (*p* = 0.0106), and Feret’s diameter (*p* = 0.0172) were significantly decreased in the 5 mM treatment group compared to controls (Fig. 1c–e). The significant decrease in area and perimeter suggests that cells treated with 5 mM PPA have smaller, rounder mitochondria and that these mitochondria are less elongated than those in control cells. This is also consistent with the significant decrease in Feret’s diameter, which is an independent parameter demonstrating a decrease in the longest distance between particle edges. Changes in the ultrastructure of cristae were observed, with cristae becoming less defined under PPA stress (Fig. 1a, panel B). However, not all images captured cristae ultrastructure with sufficient clarity; thus, these changes were not quantitatively analyzed. These TEM data may represent three possible scenarios: (1) PPA upregulates fission or downregulates fusion leading to existing mitochondria becoming smaller; (2) an increase in biogenesis produces new, smaller mitochondria or (3) both mechanisms are induced simultaneously. While these scenarios cannot be distinguished using TEM, the significant morphological changes demonstrate alterations to mitochondrial homeostasis and dynamics under PPA stress. Subsequently, we explored additional parameters to further characterize these dynamic changes and the potential underlying mechanisms.

### Propionic acid remodels mitochondrial dynamics

**Figure 2:**
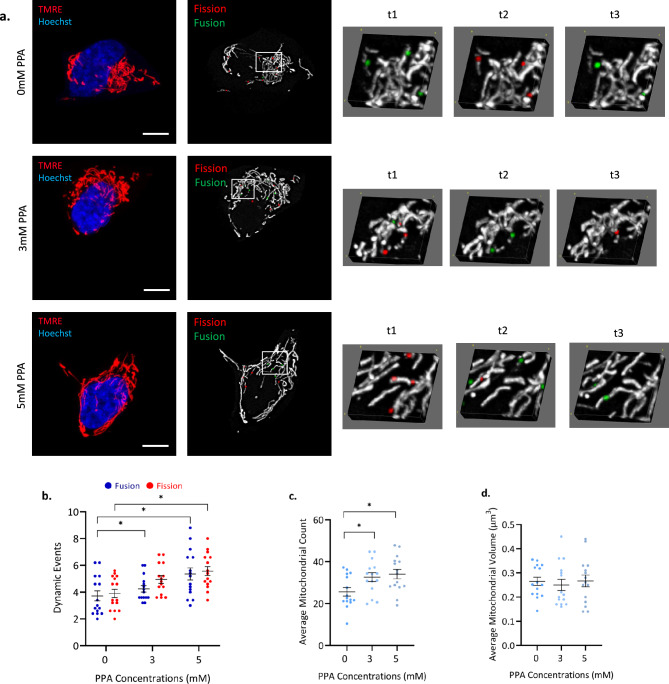
Propionic acid (PPA) leads to dynamic mitochondrial remodeling to maintain network integrity. SH-SY5Y cells were cultured, treated with 3 and 5 mM PPA for 24 h and stained using TMRE and Hoechst 33342 before by MEL analysis. (**a**) Representative images captured during time-lapse microscopy depict colored and binarized maximum intensity projections at time frame 2 (t2) for each condition. Selected regions indicated in each binary image were enhanced and shown in 3D at three different time frames (t1-t3) to illustrate the dynamic changes over time; fusion events are highlighted in green; fission events are shown in red. (**b**) Average number of dynamic events at each condition. (**c**) Average number of mitochondrial structures per cell. (**d**) Average volume per mitochondrial structure per cell (µm^3^). Data shown represent n = 15 cells per treatment group. Error bars shown represent mean ± SEM, scale bar = 10 µm, * p < 0.05.

**Figure 3:**
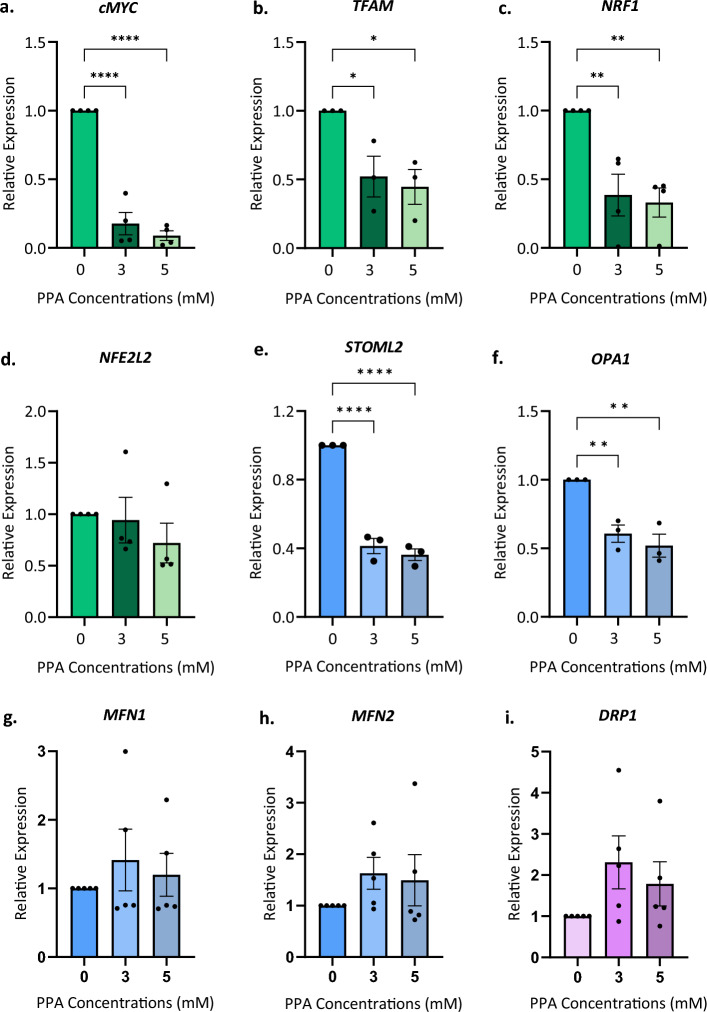
Propionic acid (PPA) induces a transcriptional downregulation of genes involved in mitochondrial dynamics. SH-SY5Y cells were treated with 3 and 5 mM PPA for 24 h. Relative quantification of genes was performed using RT-qPCR and normalized to *B2M*. Mitochondrial biogenesis genes (**a**) *cMYC*, (**b**) *TFAM,* (**c**) *NRF1*,  and (**d**) *NFE2L2.* Mitochondrial fusion and fission genes (**e**) *STOML2,* (**f**) *OPA1,* (**g**) *MFN1*, (**h**) *MFN2* and (**i**) *DRP1.* Significant differences were tested using one-way ANOVA (control compared to treatments) and Dunnett’s test for multiple comparisons (*p* < 0.05); * indicates *p* < 0.05, ** indicates *p* < 0.01, **** indicates *p* < 0.0001. Bars represent the mean expression ± SEM. The data shown represents n = 3 (*STOML2, OPA1, TFAM*), n = 4 (*cMYC, NRF1, NFE2L2*) and n = 5 (*MFN1*, *MFN2, DRP1*) biological repeats.

To further characterize how mitochondrial dynamics change in response to PPA, we stained mitochondria with tetramethyl rhodamine ethyl ester (TMRE) and used time-lapse microscopy with MEL analysis^33^ to localize and quantify mitochondrial fission and fusion events after 24 h of 3 and 5 mM PPA treatment (Fig. 2a). Following MEL analysis, mitochondria were further analyzed to quantify the numbers of mitochondrial structures, as well as their average volume. We observed a small, but significant increase in the number of fission events occurring at 3 mM [4.9 ± 0.3 (*p* < 0.05)], while both fission [5.6 ± 0.3 (*p* < 0.05)] and fusion [5.4 ± 0.5 (*p* < 0.05)] events were significantly increased at 5 mM compared to controls (Fig. 3b). The number of mitochondria significantly increased at both 3 [32.6 ± 2.1 (*p* < 0.05)] and 5 mM [34.1 ± 2.2 (*p* < 0.05)] (Fig. 3c), while the average volume per mitochondrial structure remained unchanged (Fig. 3d). Together, this indicates a remodeling of mitochondrial dynamics as a compensatory response that successfully maintains the integrity of the mitochondrial network. The increase in fission events at 3 mM PPA suggests that the increase in mitochondrial count can in part be attributed to mitochondrial fission but does not rule out biogenesis as an additional compensatory response, given that average mitochondrial volume remains largely unchanged. Nevertheless, these data are consistent with the smaller, round mitochondrial structures observed with TEM, and additionally demonstrate significant alterations to mitochondrial dynamics induced by PPA.

### Central mitochondrial regulators are perturbed by propionic acid stress

Together, TEM and MEL analysis data show that PPA alters mitochondrial morphology and dynamics. However, these visualization techniques do not provide insight into the underlying mechanisms that drive these processes. Therefore, we examined mRNA expression of nine key regulators of mitochondrial dynamics, biogenesis, and mitostasis in response to PPA treatment. We quantified gene expression of Cellular Myelocytomatosis Oncogene *(cMYC),* Nuclear Respiratory Factor (*NRF1),* Mitochondrial Transcription Factor 1 (*TFAM),* NFE2-Like BZIP Transcription Factor (*NFE2L2)*, Stomatin-Like Protein 2 (*STOML2)*, Optic Atrophy 1 *(OPA1),* Mitofusin 1 (*MFN1)*, Mitofusin 2 (*MFN2)*, and Dynamin-Related Protein 1 (*DRP1)* after 24 h treatment of 3 mM and 5 mM PPA. We observed a significant decrease in the expression of three central mitogenesis regulators, *cMYC, NRF1* and *TFAM* after 24 h of 3 mM (*p* = 0.0053, *p* = 0.0415, and *p* < 0.0001, respectively) and 5 mM (*p* = 0.0031, *p* = 0.0233, and *p* < 0.0001, respectively) PPA treatment (Fig. 3a–c). This decrease in mRNA expression followed a dose-dependent trend; *cMYC, NRF1,* and *TFAM* expression decreased by 5.7-, 2.6-, and 1.9-fold respectively at 3 mM but 11.2-, 3-, and 2.2-fold respectively at 5 mM. Conversely, the central redox-responsive biogenesis gene, *NFE2L2,* was not altered under any concentration of PPA although there was a similar dose-dependent decreasing trend in expression (Fig. 3d).

**Figure 4:**
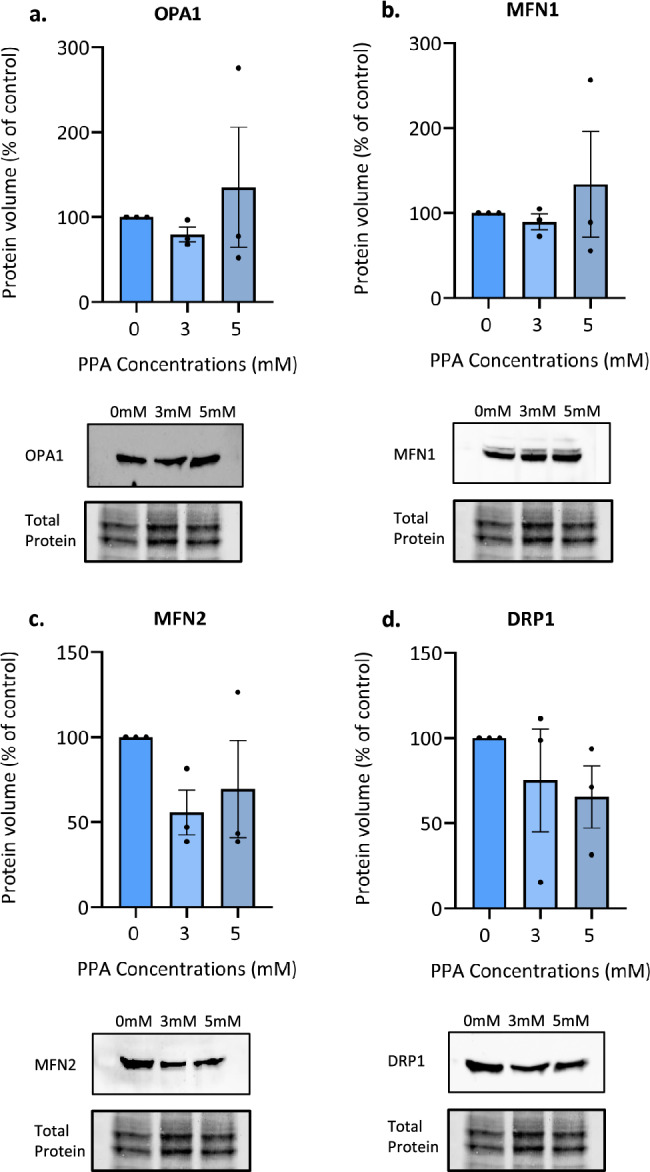
Protein levels of fusion and fission factors were unchanged after propionic acid (PPA) treatment. SH-SY5Y cells were treated with 3 and 5 mM PPA for 24 h. Protein levels were quantified using western blot analysis and expression abundance was normalized to total protein. Mean protein expression and representative western blots of the protein of interest and total protein are shown. (**a**) OPA1, (**b**) MFN1, (**c**) MFN2, and (**d**) DRP1. Bars represent mean ± SEM and data shown represents n = 3 biological repeats. Statistical analysis was performed using one-way ANOVA and Dunnett’s test for multiple comparisons (*p* < 0.05). Original gels and blots are presented in Fig. S1.

We also examined the expression of canonical genes involved in the regulation of fission and fusion. The expression of *STOML2*, which is thought to be involved in fusion, mitophagy, and biogenesis, was significantly decreased at both 3 mM (2.4-fold change) and 5 mM (2.8-fold change) PPA (*p* < 0.0001) (Fig. 3e). Similarly, the fusion gene *OPA1* had decreased expression at 3 mM (1.6-fold change) and 5 mM (1.9-fold change) PPA (*p* = 0.006 and *p* = 0.0024, respectively) (Fig. 3f). However, we found no significant differences in the expression of the fusion genes *MFN1, MFN2* or the fission gene *DRP1* under 24-h PPA stress (Fig. 3g–i). Moreover, we found that there were no changes in the protein levels of the four fusion and fission proteins (OPA1, MFN1, MFN2, and DRP1) under the same conditions (Fig. 4a–d). Importantly, these data reflect a single time point and may not reflect expression changes or levels of protein activity at earlier stages of PPA stress. Nevertheless, the significantly decreased expression of *cMYC, NRF1, TFAM, STOML2,* and *OPA1* demonstrate significant transcriptional dysregulation of mitochondrial metabolism, biogenesis, and dynamics. Moreover, these data emphasize the utility of visualization techniques to directly investigate changes to end-point mitochondrial function.

## Discussion

Mitochondrial dysfunction has been implicated in multi-systemic disorders ranging from metabolic, cardiovascular, and muscular diseases to neurological disorders^1,10^. Numerous neurodevelopmental and neurodegenerative disorders are linked to mitochondrial dysfunction, emphasizing the importance of these organelles throughout the brain’s lifespan. These disorders include Parkinson’s and Alzheimer’s disease and ASD^3,4,18^. However, it is difficult to access brain tissue to study these diseases, particularly at the mechanistic level, thus cell model systems serve as a necessary alternative. In this study, we utilized a cell model system using SH-SY5Y cells treated with PPA to recapitulate mitochondrial dysfunction seen in neuronal disorders, specifically ASD. Using this PPA model to examine mitochondrial dynamics in neurons may yield insight into ASD etiology.

We explored the feasibility of using TEM to view mitochondrial morphological changes. Importantly, TEM must be used appropriately to maximize its utility. Cryogenic sample preparation allows for the simultaneous immobilization of cellular components and decreased formation of artifacts, which together better preserve neuronal architecture^34^. Consistent with this, we observed that neuronal-like SH-SY5Y cells displayed intact subcellular organelles and elongated mitochondria (Fig. 1a). This highlights the utility of cryogenic preparation approaches to study mitochondrial morphology in neuronal cell models. While it is increasingly evident that quantitative measures are essential for the objective analysis of TEM data, there is still no consensus regarding which specific parameters should be measured to confirm mitochondrial morphological changes. Based on numerous studies that quantitatively examined mitochondrial morphology^17,31,32^, we developed an automated mitochondrial image analysis pipeline that measured eight morphological parameters, namely, area, area^2^, form factor, aspect ratio, perimeter, circularity, Feret’s diameter and roundness.

Of these, PPA significantly decreased area^2^, area, perimeter and Feret’s diameter (Fig. 1b–e). This suggests that mitochondria became smaller and rounder, which is consistent with previous studies showing that mitochondrial area decreased after 72 h of PPA-induced mitochondrial stress^30^. These morphological features may be indicative of mitochondrial fission, a process essential for separating damaged components from the mitochondrial network to facilitate their degradation by mitophagy^35–37^. On the other hand, a decrease in average mitochondrial size can result from the upregulation of biogenesis, which produces small, nascent mitochondria. An increase in either fission or biogenesis represents compensatory responses to maintain mitostasis in response to mitochondrial stress. However, reduced mitochondrial growth, impaired fusion, or other scenarios cannot be excluded.

While TEM produces high resolution images that allow morphological characterization at the level of single mitochondria, this method produces a two-dimensional snapshot at a single time point. To investigate dynamic responses to metabolic stress, we stained mitochondria with TMRE and employed time-lapse microscopy with MEL analysis which allows for high-throughput, three-dimensional visualization of mitochondrial networks over time^33,38^. We observed subtle but significant changes to mitochondrial dynamics under PPA stress (Fig. 2). At 3 mM, there is a significant increase in fission events, while fusion events remain similar to the controls. An increase in both fission and fusion events is observed at 5 mM PPA, however these changes are approximately proportional, suggesting equilibrium of fission and fusion dynamics is reached at this higher concentration (Fig. 2b). Average mitochondrial volume remained unchanged at both 3 and 5 mM PPA which illustrates the maintenance of mitochondrial network integrity (Fig. 2d). This reflects a dynamic mitochondrial network with the capacity to respond to mild metabolic stress to effectively maintain homeostasis without leading to network fragmentation. At 3 mM PPA, an increase in fission is sufficient to facilitate a transition to a new equilibrium, but more profound remodeling of dynamics is required to respond to the stress induced at higher concentrations of PPA.

Mitochondrial count increases at both concentrations of PPA stress, without significant changes to average mitochondrial volume (Fig. 2c). This could result from either increased biogenesis or increased fission; however, an increase in biogenesis is more likely in the absence of a significant decrease in mean mitochondrial volume. Nevertheless, the data in Fig. 2 does provide evidence for both mechanisms of compensation: the increase in the number of fission events is congruent with an upregulation of mitochondrial fission while the increase in count is consistent with mitochondrial biogenesis. Ultimately, the dynamic compensation in response to mild stress is likely to consist of simultaneous processes involving fission, fusion, biogenesis, and mitophagy. While previous authors have shown that PPA upregulates mitogenesis^30,39^ and mitophagy^29^, we provide evidence for a remodeling of mitochondrial fission and fusion dynamics in response to PPA. This data supports the morphological changes observed using TEM and provides further insight into the mechanisms associated with PPA-induced mitochondrial dysfunction.

Since neither TEM nor MEL analysis provides direct evidence about which gene regulatory mechanisms underlie the morphological changes observed, we examined RNA expression of genes involved in mitochondrial metabolism, biogenesis, and dynamics. The cMYC proto-oncogene is a transcription factor that is involved in the regulation of mitochondrial, glycolytic, amino acid, and fatty acid metabolism^40^. Moreover, *cMYC* is known to regulate the expression of almost 600 mitochondrial genes involved in mitochondrial transcription, translation, and complex assembly, including *NRF1* and *TFAM*^41^*. NRF1* and *TFAM* are two central regulators of mitogenesis that function downstream of PGC-1α to upregulate mtDNA replication. This pathway is activated by cAMP and AMPK signaling, which is sensitive to energy depletion and metabolic stress. We also examined *NFE2L2*, which is a redox-responsive regulator of mitochondrial biogenesis, to determine whether the effects of PPA may be mediated by oxidative stress.

While *NFE2L2* expression remained unchanged, we found a consistent dose-dependent decrease in *cMYC, NRF1,* and *TFAM* expression after 24 h at 3 mM and 5 mM PPA treatment (Fig. 3a–c). Decreased *cMYC* expression was previously reported in response to mitochondrial stress^42^ and conversely, downregulating *cMYC* was shown to induce mitochondrial dysfunction by reshaping mitochondrial metabolism, network connectivity and membrane polarization^43^. Interestingly, cMYC is also implicated in the regulation of mitochondrial fission and fusion^42,43^ and is known to increase DRP1 phosphorylation and mitochondrial localization during cell division^44^, as well as mediate mitochondrial morphological remodeling in neuronal stem cells^45^. In fact, cMYC-null fibroblasts displayed decreased mitochondrial size which is consistent with the changes induced by PPA stress^43^. These data illustrate an intriguing, but still poorly characterized, relationship between *cMYC* and mitochondrial dynamics that presents an interesting target for future research into the remodeling induced under PPA stress.

The decrease in *NRF1* and *TFAM* are in line with the role played by cMYC as an important transcriptional activator. These data are also congruent with previous studies in human colon cancer cells showing that PPA decreases *NRF1* mRNA expression after 22 h, which was associated with ATP depletion and increased ROS^46^. These authors also reported that *TFAM* expression increased at 8.5 h but returned to baseline levels after 22 h. In contrast, Kim et al. (2019) showed a significant decrease in *TFAM* mRNA expression in SH-SY5Y cells after 4 h of PPA stress; however, TFAM protein expression was significantly upregulated and mtDNA copy number significantly increased after 72 h. Therefore, the decrease in mitochondrial biogenesis genes we observe at 24 h does not exclude the possibility that the increase in mitochondrial count is due to an upregulation of biogenesis at earlier time points. Previous work has shown that PPA significantly upregulates PGC-1α mRNA and protein in SH-SY5Y cells after 4 h^30^ while propionate increased mitochondrial biogenesis via PGC-1α after 12 h in calf hepatocytes^39^. Interestingly, PGC-1α is not only a direct transcriptional regulator of both *NRF1* and *TFAM* but has also been shown to regulate MFN2 and DRP1 activity to modulate fission and fusion^47^. Together, this highlights the tight coupling of mechanisms that regulate the mitochondrial compensation responses induced by PPA. Moreover, our data reflect significant perturbations to the transcriptional regulation of biogenesis and metabolism under PPA stress.

The genes *STOML2, OPA1, MFN1*, *MFN2,* and *DRP1* are some of the central regulators of mitochondrial fission, fusion, and dynamics^37,48,49^. There are many additional genes implicated in mitochondrial dynamics; however, *STOML2, OPA1,* and *MFN2* were previously found to be differentially methylated in an ASD cohort^16^ while several independent studies reported alterations to these transcription factors in response to mitochondrial stress^50–52^. The expression of both *OPA1* and *STOML2* was significantly decreased at 3 mM and 5 mM PPA treatment (Fig. 3e,f). OPA1 is one of the canonical regulators of mitochondrial fusion via direct interactions with MFN1 and 2 and plays a role in the remodeling of cristae and mitochondrial morphology^53^. The precise role of *STOML2* in mitochondrial dynamics remains elusive, but evidence suggests that it plays a role in mitochondrial fusion, biogenesis and mitophagy.

STOML2 is involved in maintaining coupled mitochondrial respiration and the formation of respiratory chain complexes^54,55^ and has been shown to profoundly alter the metabolic profile of cancer cells^56^. STOML2 was found to promote mitochondrial membrane potential and biogenesis via interactions with prohibitions and cardiolipin^55,57,58^. Moreover, independent studies have demonstrated an interaction between STOML2 and PINK1 that modulates mitophagy^59,60^. Notably, it has been reported that STOML2 directly interacts with and stabilizes MFN2 and plays an important role in stabilizing the long OPA1 isoform by inhibiting the protease responsible for OPA1 cleavage^53,61,62^. The decrease in *STOML2* expression observed in response to PPA may render these fusion proteins more susceptible to degradation by ubiquitin and proteasome-dependent pathways^48^. While the precise role of STOML2 and OPA1 in the dynamic response to PPA is unclear, it is possible that the decreased expression of these fusion genes (Fig. 3) disrupts the balance between fission and fusion and contributes to the decrease in mitochondrial size (Fig. 1).

On the other hand, protein expression of OPA1 remained unchanged after 24 h, while neither mRNA nor protein levels of MFN1, MFN2, or DRP1 were significantly altered by PPA treatment (Fig. 3g–i, Fig. 4). This could indicate that there are no changes in the regulation of these factors involved in mitochondrial fusion and fission. However, it is important to note that each of these four genes is also regulated by post-transcriptional modifications (PTMs) that control protein activity. OPA1 has eight alternative splice variants which are proteolytically cleaved in the mitochondria to produce two different isoforms^63^. It is the balance between long and short isoforms that ultimately determines the role played by OPA1 in mitochondrial fusion and mitochondrial network maintenance^64^. DRP1 activity is regulated by phosphorylation by calcium/calmodulin-dependent protein kinase-II (CaMKII), while DRP1 degradation is regulated by ubiquitylation and SUMOylation^65^. Finally, both DRP1 and MFN1/2 are GTPases, thus activity may be influenced by the rate of GTP production in the mitochondria^66^. Therefore, while the expression of these proteins remained constant, this may not reflect unaltered protein activity or localisation^67,68^. In fact, PTMs of an existing pool of proteins often serve as the first line of defense that are responsible for carrying out acute stress responses. In the presence of the mild metabolic stress induced in our model, PTMs may well facilitate an increase in the activity of fusion and fission proteins to sufficiently rescue mitochondrial integrity, without requiring an additional upregulation of these genes on the mRNA or protein level.

Collectively, the data presented above highlights the complex and temporally specific regulation of mitochondrial morphology, and the challenges of unraveling these mechanisms. To examine gene expression, specific target genes in a pathway first need to be identified. However, our data show that genes within the same pathway do not respond homogeneously to the same stress. In fact, previous studies have shown that different genes in the same pathway can display different temporal response profiles^30,46^. In addition, there are complex post-transcriptional mechanisms that confound the relationship between gene transcription and function. Proteomic studies may yield some insight into the effect of PTMs and protein function, but this also presents challenges, including low-throughput methods, high signal-to-noise ratios, and poor resolution.

In this context, using TEM and MEL to study mitochondrial morphology has great potential to address fundamental questions about the relationship between mitochondrial dynamics and function, and how this impacts disease. Most significantly, TEM provides a direct way to measure mitochondrial morphology, which serves as a convergent endpoint parameter for disruptions to mitochondrial function and dynamics^51^. MEL further provides a direct way to visualize fission and fusion events in the three-dimensional cellular context, allowing for the quantification of dynamic mitochondrial remodeling even in the absence of changes in gene expression^33^. Here, we highlight the utility of mitochondrial visualization techniques in the context of secondary mitochondrial disorders. These disorders are often characterized by chronic mild metabolic stress, marked by subtle remodeling of the mitochondrial network rather than acute mitochondrial damage. Nevertheless, the mitochondrial compensation required to maintain mitostasis under chronic stress has profound functional consequences. In a neuroscience context, a better understanding of these compensatory mechanisms could yield important insight into the pleiotropic neuropathologies that are associated with mitochondrial dysfunction.

Ultimately, our data highlight the utility of imaging techniques to provide insight into the functional outcome of the complex interplay between gene expression, protein modifications, and protein activity that governs mitochondrial dynamics in neurons. We used PPA to model mitochondrial dysfunction in a neuronal cell model to yield insight into the mitochondrial component of ASD. SH-SY5Y cells treated with PPA displayed altered mitochondrial morphology with small, round mitochondria, with poorly defined cristae when visualized using TEM. MEL analysis showed that these changes occurred in conjunction with an increase in fission and fusion events to maintain the mitochondrial network in response to mild metabolic stress. Moreover, PPA significantly disrupted the transcriptional regulation of mitochondrial metabolism and homeostasis. We identified *cMYC, NRF1, TFAM*, *STOML2* and *OPA1* as key mitochondrial regulators perturbed by PPA stress that may play a role in mediating PPA-induced changes to mitochondrial morphology and function. Future studies are needed to better characterize temporal changes to gene expression and protein activity, localization, and post-translational modifications induced by PPA. Our data highlight the complex and interdependent nature of the regulatory mechanisms that mediate responses to mitochondrial stress and demonstrate the utility of TEM and other visualization techniques to inform more targeted mechanistic investigations.

## Methods

### Cell culture

The SH-SY5Y cell line (ECACC, 94,030,304-1VL) was purchased from Sigma-Aldrich. SH-SY5Y cells were grown in 25 cm^2^ flasks in Dulbecco's Modified Eagle Medium/Nutrient Mixture F-12 (DMEM/F-12) with L-Glutamine (SC09411, ScienCell) supplemented with 20% Fetal Bovine Serum (FBS) (10493106, ThermoFisher Scientific) and 1% Penicillin–Streptomycin (P4333-20ML, Sigma-Aldrich) at 37 °C, 5% CO_2_. Cells were sub-cultured to 80% confluency using 0.05% Trypsin–EDTA (15400054, ThermoFisher Scientific), centrifuged at 300*g* and seeded at approximately 7 × 10^5^ cells/ml. All experiments were done on undifferentiated SH-SY5Y cells between passages 19–22. PPA was administered in the form of NaP. NaP powder (CAS number 137-40-6, chemical formula C_3_H_5_NaO_2,_ P5436-100G, Sigma-Aldrich) was dissolved in warm MilliQ water to a concentration of 1 M and stored at 4 °C. This 1 M PPA solution was diluted in serum-free media (DMEM/F-12 with L-Glutamine) on the day of treatment to 3 mM and 5 mM PPA. Treatment concentrations for all experiments were no PPA (0 mM, control), 3 mM, and 5 mM PPA. Experiments were done in a minimum of three biological replicates.

### Transmission electron microscopy

#### Cryogenic sample preparation

SH-SY5Y cells were seeded at 5.5 × 10^5^ cells/ml in 25 cm^5^ flasks and grown for 24 h. PPA treatments were added to the flasks before incubating for 24 h. Routine mammalian tissue sub-culturing protocol (described above) was followed to collect cell pellets. The cell pellets were resuspended in 100 µl 2.5% glutaraldehyde, 1 × PBS, and stored at 4 °C until processing. SH-SY5Y cells were briefly centrifuged to pellet the cells and remove the 2.5% glutaraldehyde, 1 × PBS solution. Pellets were resuspended in 4% agarose gel made with distilled water (1:1 ratio agarose to pellet volume). Agarose slices were placed onto grids on a flat planchette and covered with 1-hexadecene before high-pressure freezing. Samples were freeze substituted at − 90 °C for 24 h in 100% dry acetone. This was followed by raising the temperature to − 80 °C and adding a solution of 1% osmium tetroxide and 0.1% glutaraldehyde. Samples were kept at − 80 °C for 24 h. Following this, the temperature gradually increased to room temperature over multiple days: from − 80 °C to − 50 °C for 24 h, to − 30 °C for 24 h, to − 10 °C for 24 h and finally to room temperature.

#### Infiltration, staining, and microscopy

After cryogenic sample preparation, samples were infiltrated with resin, and ultrathin sections (∼100 nm) were cut with a Leica Reichert UltracutS Ultramicrotome (Leica Microsystems). Sections were stained with 2% uranyl acetate and lead citrate. Samples were viewed using a FEI Tecnai 20 transmission electron microscope (ThermoFisher (formerly FEI), Eindhoven, Netherlands) operating at 200 kV (Lab6 emitter) and fitted with a Tridiem energy filter using a Gatan CCD camera (Gatan, UK).

#### Image analysis

A minimum of 24 images of individual cells were captured per technical replicate with a total of 266 images overall. The region of interest (ROI) macro and the mitochondrial macro were used to analyze all images. The mitochondrial macro was based on published methods^17,31,32^ and allowed for the semi-automated batch processing of TEM images in Fiji/ImageJ^69^. Briefly: Images were inverted and preprocessed using a rolling ball background subtraction (radius of 60 pixels) and a FFT bandpass filter using upper and lower bounds of 60 and 8 pixels, respectively, and vertical line suppression with 5% tolerance of direction. The processed images were automatically thresholded using the maximum entropy algorithm and binary masks were generated. Image regions were extracted correlating with manually selected ROIs in unprocessed TEM images, featuring mitochondria and excluding plasma membrane and other regions of high contrast. For each extracted ROI, binary particles greater than 600 pixels were analyzed and particle area, perimeter, major and minor axes, Feret's diameter, circularity, and roundness were measured using the Fiji/ImageJ built-in measurement function. From these, area^2^, particle aspect ratio (the ratio of major and minor axis), and form factor (FF), where FF = perimeter^2^/4*pi x* area, as per Merrill, Flippo and Strack (2017), were calculated. Definitions for the formulae of the parameters can be found in Merrill, Flippo and Strack (2017). The macros mentioned are available on GitHub (see the data availability statement). On average, approximately 5600 particles were analyzed for each PPA treatment, and approximately 17,000 particles were analyzed overall (data not shown).

### Mitochondrial event localiser (MEL)

The SH-SH5Y cells were plated in 8-chamber dishes (ThermoFisher, #155411) to allow overnight adhesion before being stained with 1:1000 TMRE (ThermoFisher, #T669) and 1:200 Hoechst 33342 (Sigma-Aldrich, H6024) in media for 10 min. Images were captured using 405 nm and 561 nm lasers and raw images were acquired as z-stacks containing 10 image micrographs with a z-step width of 0.2 µm between image frames over 12 subsequent time points. Images were acquired using a Carl Zeiss LSM780 ELYRA PS.1 Super-resolution platform (Carl Zeiss, Oberkochen, Germany) using a LCI Plan Apochromat 100x/1.4 Oil DIC M27 objective. Images were analyzed in ImageJ using a previously described pipeline and the ImageJ plugin to measure fusion and fission events, average mitochondrial structure count and average mitochondrial volume per cell^33^. The MEL macro is available on GitHub (see the data availability statement).

### RNA and protein extractions

The SH-SY5Y cells were grown in a six-well plate at a density of 0.3 × 10^6^ cells/ml for 24 h before treatment. RNA was extracted using the Quick-RNA™ Miniprep (ZR R1055, Zymo Research) protocol with minor modifications: 300 µl of RNA lysis buffer was added to each well before scrapping and in the final step each sample was eluted in 30 µl DNase/RNase-free water. The quantity and quality of all samples was checked using the NanoDrop ND-1000 UV–Vis Spectrophotometer. Total protein from cell lysates were prepared using 200 µl of RIPA lysis buffer and protein concentration was quantified using the Bradford protein assay^70^.

### Quantitative real-time PCR (RT-qPCR)

The cDNA synthesis was performed using the Tetro™ cDNA Synthesis Kit (BIO-65043, Meridian Bioscience) following the manufacturer’s instructions with some modifications. Total RNA between 0.7 and 1 μg was used to synthesize cDNA in a 20 μl reaction. Primers were selected from previously published papers^42,71–78^ (Table S1) and their accompanying probes were designed using the Integrated DNA Technologies’ PrimerQuest Tool. All genes of interest were normalized to the nuclear gene *B2M*. Gene expression of *STOML2, NRF1, NFE2L2*, *TFAM, cMYC* and *OPA1* was measured using RT-qPCR. The master mix included LUNA Taq polymerase (M3003L, New England Biolabs), 10 µM forward and reverse primers, cDNA, and PCR-grade water to make up a final volume of 10 µl per reaction. Gene expression of the fission and fission genes (*DRP1*, *MFN1*/*2*) were measured in a multiplex TaqMan assay. Luna Universal Probe qPCR Master Mix (M3004S, New England Biolabs) was used according to the manufacturer’s instructions with minor modifications. The multiplex RT-qPCR master mix included 1X LUNA Taq polymerase, 10 µM of forward and reverse primers, 10 µM of probes, cDNA, and PCR-grade water to make up a final volume of 20 µl per reaction. Rotor-Gene Q 6-plex (QIAGEN RG—serial number: R0618110) was used to perform RT-qPCR and cycling conditions can be found in Table S1. All cDNA samples were amplified in triplicate with a standard curve in tenfold dilution series. Outliers within the triplicate samples with a cycle threshold (Ct) standard deviation > 0.5 were removed from the analysis to ensure data reproducibility^30,72^. Relative gene expression was calculated using the 2^−ΔΔCt^ method^79^.

### Western blot analysis

The protein samples (60 µg) were mixed with Laemmli loading buffer at a 2:1 ratio and electrophoresed on 12% stain free protein gels (Bio-Rad #1610184). Proteins were transferred to a PVDF (polyvinylidene difluoride) membrane (#170-84156, Bio-Rad) using the Trans-Blot Turbo system (#170-4155, Bio-Rad). The membranes were blocked and incubated with relevant primary antibodies (OPA1, MFN1, MFN2 and DRP1) (diluted 1:1000) for 48 h, followed by incubation with the secondary antibody (1:10 000) for 1 h. Membranes were then imaged using Clarity Western ECL substrate (#170-5061, Bio-Rad) and acquired using the Bio-Rad ChemiDoc MP System. ImageLab version 6.1 was used to analyze western blots. Original gels and blots are presented in Fig. S1. Information about the antibodies is provided in Table S2.

### Statistical analysis

Data sets are shown as the mean and standard error of the mean (SEM) of at least three independent samples. Data sets were tested for normality using the Shapiro-Wilks test before assuming Gaussian distribution and equal standard deviations and proceeding with the analysis (unless otherwise stated). One-way ANOVA (mean of treatments compared to controls) and Dunnett’s test for multiple comparisons were used to determine significance (*p* < 0.05) aside from data sets from MEL analyses where Fisher’s LSD was used (*p* < 0.05). Significant p values are shown in graphs as *p < 0.05, **p < 0.01, ***p < 0.001, ****p < 0.0001. All statistical analyses and graphs were performed and generated using GraphPad Prism 9.4.0.

### Supplementary Information

Supplementary Information.

## Data Availability

The Fiji/ImageJ macros used for TEM image analysis are publicly available on GitHub here: https://github.com/caaja/TEMMitoMacro. The mitochondrial event localiser (MEL) macro is publicly available on GitHub here: https://github.com/rensutheart/MEL-Fiji-Plugin.
