# Supplementary material for: Propionic acid induces alterations in mitochondrial morphology and dynamics in SH-SY5Y cells
Source: Sci Rep. 2023 Aug 15;13:13248. doi: 10.1038/s41598-023-40130-8 (PMC10427685; doi:10.1038/s41598-023-40130-8)
Supplement: Supplementary file 1 — Supplementary Information. [file 41598_2023_40130_MOESM1_ESM.pdf]

Supplementary Table S1: Primer and probe sequences and conditions

Gene	Primer sequence (5'-3')		Probe sequence (5'-3')	Fluorescent dye:Quencher	Cycling Conditions	Reference
cMYC	Forward	ACCACCAGCAGCGACTCTGA	N/A	N/A	95°C for 60s;<br>40 cycles of 95°C for 10s, 60°C for 30s, 72°C for 10s;<br>with melt curve.	Wang et al., 2018
	Reverse	TCCAGCAGAAGGTGATCCAGACT				
DRP1	Forward	TCAACCTCCGCGTCTACTC	CGTGCTGAACCTGACCCTGGT	FAM:BHQ-1	95°C for 60s;<br>40 cycles of 95°C for 15s, 63°C for 30s and 72°C for 10s;<br>with melt curve.	Kosmider et al., 2019
	Reverse	GATCTGGAACTCGATGTCGGG				
MFN1	Forward	GAGGTGCTATCTCGGAGACAC	GGCAGGACAAGCAGTGGGAAGA	Cy5:BMN-Q620 3'	95°C for 60s;<br>40 cycles of 95°C for 15s, 63°C for 30s, 72°C for 10s;<br>with melt curve.	
	Reverse	GCCAATCCCACTAGGGAGAAC				
MFN2	Forward	TGGCTCAAGACTATAAGCTGCG	AGATTACGGAGGAAGTGGAGAGGCA	HEX:BHQ-1	95°C for 60s;<br>40 cycles of 95°C for 15s, 63°C for 30s, 72°C for 10s;<br>with melt curve.	Duraisamy, Mohammad and Kowluru, 2019
	Reverse	GAGGACTACTGGAGAAGGGTGG				
NFE2L2	Forward	GAGAGCCCAGTCTTGGAAC	TCGCTCAGTTACAACTAGATGAAGAGA	HEX:BHQ-1	95°C for 60s;<br>40 cycles of 95°C for 10s, 60°C for 30s,72°C for 10s;<br>with melt curve.	Schrier et al., 2022
	Reverse	TTGGCTTCTGGACTTGGAAC				
NRF1	Forward	TTGAGTCTAATCCATCTATCCG	TCGGAAACTTCGAGCCACGTTAGA	Cy5:BMN-Q620 3'	95°C for 60s;<br>40 cycles of 95°C for 10s, 60°C for 30s;<br>72°C for 10s; with melt curve.	Pinho et al., 2022
	Reverse	TACTTACGCACCACATTCTC				
OPA1	Forward	AGCCTCGCAGGAATTTTTGG	N/A	N/A	95°C for 60s;<br>40 cycles of 95°C for 10s, 60°C for 40s, 72°C for 20s;<br>with melt curve.	Bean et al., 2021
	Reverse	AGCCGATCCTAGTATGAGATAGC				
TFAM	Forward	GTCACTGCCTCATCCACC	AAGGTCTGGAGCAGAGCTGTGC		95°C for 60s;<br>40 cycles of 95°C for 10s, 60°C for 30s, 72°C for 10s,<br>with melt curve.	Ye et al., 2022
	Reverse	CCGCCCTATAAGCATCTT				
STOML2	Forward	GTGACTCTCGACAATGTAAC	N/A	N/A	95°C for 10 min;<br>40 cycles of 95°C for 15s, 62°C for 30s;<br>with melt curve.	Hu et al., 2018
	Reverse	TGATCTCATAACGGAGGCAG				
B2M	Forward	CCAGCAGAGAATGGAAAGTCAA	ATGTGTCTGGGTTTCATCCATCCGACA	CAL Fluor red 610:BHQ-2	Conditions according to gene of interest	Grady et al., 2014
	Reverse	TCTCTCTCCATTCTTCAGTAAGTCAACT				

Supplementary Table S2: Additional information for antibodies

Protein	Antibody	Source
DRP1	DRP1 (D6C7) Rabbit mAb #8570	Cell Signalling
MFN1	Mitofusin-1 (D6E2S) Rabbit mAb #14739	Cell Signalling
MFN2	Mitofusin-2 (D1E9) Rabbit mAb #11925	Cell Signalling
OPA1	OPA1 (D7C1A) Rabbit mAb #67589	Cell Signalling
HRP-linked anti-rabbit secondary antibody	Anti-rabbit IgG, HRP-linked Antibody #7074	Cell Signalling

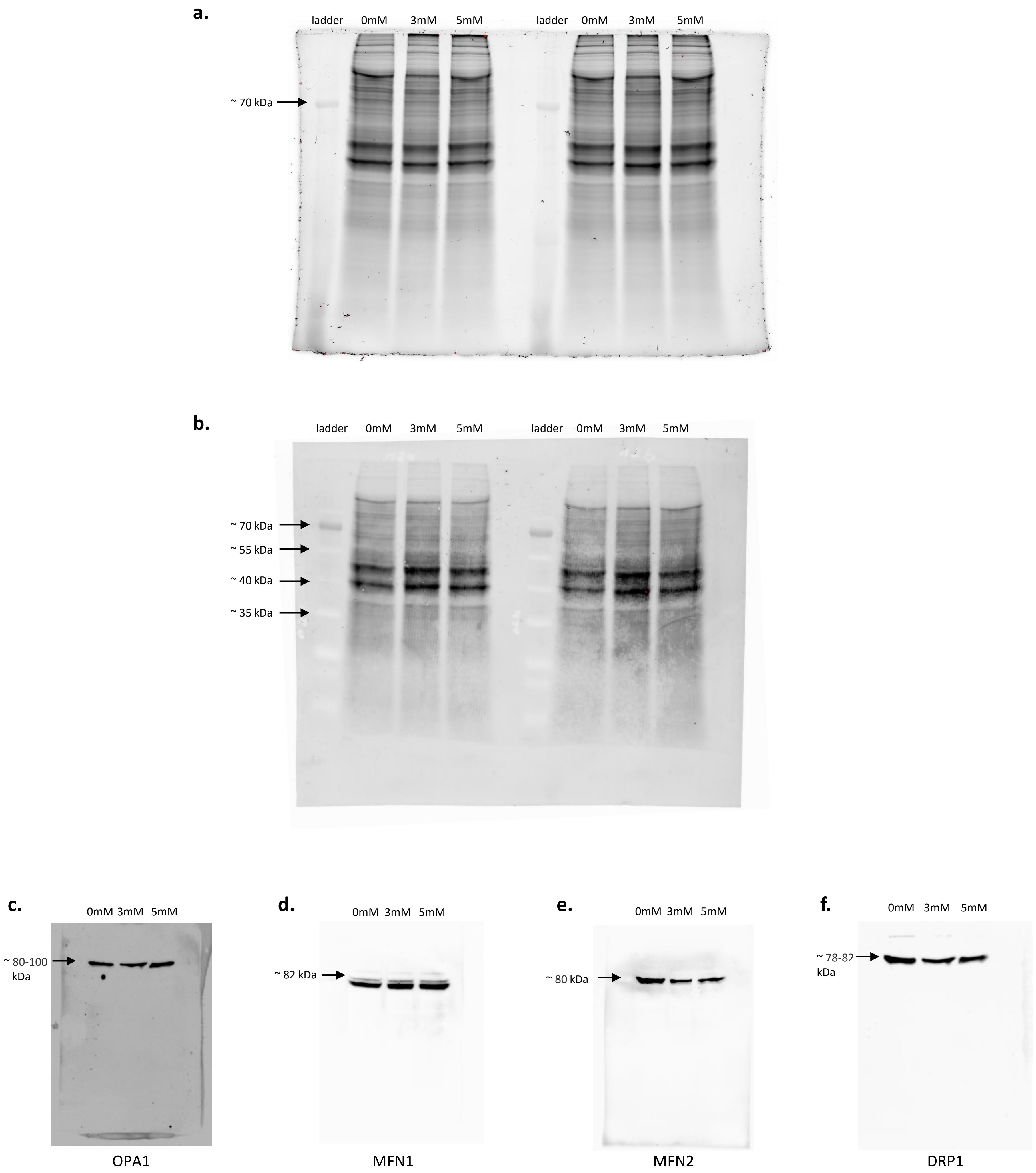

Supplementary Figure 1: Representative gels, stain-free image of a PVDF (Polyvinylidene difluoride) membrane, and western blots for proteins OPA1, MFN1, MFN2 and DRP1. SH-SY5Y cells were treated with 3 and 5 mM PPA for 24 hours. Densitometric analysis of western blots was used to analyse protein abundance which was normalized to the total protein of the control lane. **a.** Representative stain-free 10-well acrylamide gel image post electrophoresis. **b.** Representative stain-free blot image showing total protein post electrophoretic transfer. Chemiluminescence images of **c.** OPA1, **d.** MFN1, **e.** MFN2 and **f.** DRP1. Data shown is representative of n = 3 biological repeats.
